# Arabidopsis plasma membrane H^+^-ATPase genes *AHA2* and *AHA7* have distinct and overlapping roles in the modulation of root tip H^+^ efflux in response to low-phosphorus stress

**DOI:** 10.1093/jxb/erx040

**Published:** 2017-03-28

**Authors:** Wei Yuan, Dongping Zhang, Tao Song, Feiyun Xu, Sheng Lin, Weifeng Xu, Qianfeng Li, Yiyong Zhu, Jiansheng Liang, Jianhua Zhang

**Affiliations:** 1 Key Laboratory of Ministry of Education for Genetic Breeding and Multiple Utilization of Crops, Fujian Agriculture and Forestry University, Fuzhou 350002, China; 2 School of Life Sciences and State Key Laboratory of Agrobiotechnology, The Chinese University of Hong Kong, Hong Kong; 3 Yangzhou University, Jiangsu, China; 4 Nanjing Agricultural University, Nanjing, China

**Keywords:** Arabidopsis, low phosphorus, plasma membrane H^+^-ATPase, proton flux, root growth, root hair.

## Abstract

Phosphorus deficiency in soil is one of the major limiting factors for plant growth. Plasma membrane H^+^-ATPase (PM H^+^-ATPase) plays an important role in the plant response to low-phosphorus stress (LP). However, few details are known regarding the action of PM H^+^-ATPase in mediating root proton (H^+^) flux and root growth under LP. In this study, we investigated the involvement and function of different Arabidopsis PM H^+^-ATPase genes in root H^+^ flux in response to LP. First, we examined the expressions of all Arabidopsis PM H^+^-ATPase gene family members (*AHA1*–*AHA11*) under LP. Expression of *AHA2* and *AHA7* in roots was enhanced under this condition. When the two genes were deficient in their respective Arabidopsis mutant plants, root growth and responses of the mutants to LP were highly inhibited compared with the wild-type plant. *AHA2*-deficient plants exhibited reduced primary root elongation and lower H^+^ efflux in the root elongation zone. *AHA7*-deficient plants exhibited reduced root hair density and lower H^+^ efflux in the root hair zone. The modulation of H^+^ efflux by *AHA2* or *AHA7* was affected by the action of 14-3-3 proteins and/or auxin regulatory pathways in the context of root growth and response to LP. Our results suggest that under LP conditions, *AHA2* acts mainly to modulate primary root elongation by mediating H^+^ efflux in the root elongation zone, whereas *AHA7* plays an important role in root hair formation by mediating H^+^ efflux in the root hair zone.

## Highlight

Two plasma membrane H^+^-ATPases, encoded by *AHA2* and *AHA7*, enhance primary root elongation and root hair development by mediating H^+^ efflux in the elongation and root hair zones under low-phosphorus stress.

## Introduction

Phosphorus is one of the most important nutrients for plant growth, development, and reproduction. Even when the phosphorus content of soil is high, phosphorus deficiency can arise due to mineralization and fixation processes. Thus, low-phosphorus stress (LP) is one of the major limiting factors for plant growth in many ecosystems and a major constraint for agricultural productivity (Devaiah *et al.*, 2007; Schachtman and Shin, 2007). In order to survive, plants have developed flexible strategies to cope with low-phosphorus soil by improving phosphorus mobilization and uptake from the soil (Vance *et al.*, 2003; Wu *et al.*, 2003; Hoffland *et al.*, 2006; Li *et al.*, 2009). These strategies include the production of enzymes, such as acid phosphatases and nucleases; increased synthesis and secretion of organic acids or protons (H^+^) in the roots; enhanced expression of high-affinity phosphorus transporters; development of a larger root system capable of exploring a greater soil volume to ensure a sufficient uptake and use of phosphorus; and establishment of differential photosynthate distribution between shoots and roots, resulting in prolific root growth and increased root/shoot ratios. Among these strategies, plant plasma membrane H^+^-ATPase (PM H^+^-ATPase) plays the key role in the response to LP. Phosphorus acquisition and utilization are a highly regulated and complex set of processes relying not only on PM H^+^-ATPase-dependent H^+^ release, but also on PM H^+^-ATPase regulatory pathways controlling the abundance and activity of transporters and enzymes involved in phosphorus deficiency responses (Yan *et al.*, 2002).

In plants, PM H^+^-ATPases constitute a family of proton pumps driven by ATP hydrolysis. Their role is to provide an energy source for the transport of nutrients into the plant cell by generating an electrochemical gradient (Haruta and Sussman, 2012). Most of the membrane-bound transport proteins in plants are driven indirectly by the action of PM H^+^-ATPase (Palmgren, 2001). PM H^+^-ATPases form a large family of proteins in plants. For example, in Arabidopsis, there are 11 functional PM H^+^-ATPase family members, denoted *AHA1* to *AHA11* (for *A*rabidopsis *H*^+^-*A*TPase). Of the 11 genes encoding functional PM H^+^-ATPase proteins in plants, the first to be cloned were *AHA1* and *AHA3*. Using genetic analysis, roles for *AHA1* in steroid signaling, *AHA2* in iron transport, *AHA3* in pollen development, *AHA4* in salt stress, and *AHA10* in vacuole biosynthesis have been reported (Robertson *et al.*, 2004; Baxter *et al.*, 2005; Gévaudant *et al.*, 2007; Santi and Schmidt, 2009; Caesar *et al.*, 2011). Using RT-PCR, Ueno *et al.* (2005) determined that eight AHA genes are expressed in green leaves (*AHA1*, *2*, *3*, *5*, *7*, *8*, *10*, and *11*), eight are expressed in roots (*AHA1*, *2*, *3*, *4*, *7*, *8*, *10*, and *11*), and three are expressed in all tissues (*AHA1*, *2* and *11*).

Plant PM H^+^-ATPases are regulated by a number of factors, including blue light, fusicoccin, protein kinase, 14-3-3 proteins, and auxin (Palmgren, 2001; Hager, 2003; Rodrigues *et al.*, 2014). The C-terminus of plant PM H^+^-ATPases serves as an autoinhibitory domain to regulate enzymatic activity. The regulatory C-terminal domain of PM H^+^-ATPases is phosphorylated *in vivo*. *In vitro*, the domain becomes phosphorylated within seconds of exposure to blue light and fusicoccin. Phosphorylation of PM H^+^-ATPase Thr-947 by a protein kinase generates a binding site for 14-3-3 proteins. Activated PM H^+^-ATPases consist of six phosphorylated PM H^+^-ATPase molecules, assembled in a hexameric structure, together with six 14-3-3 molecules. Auxin also regulates PM H^+^-ATPase activity by phosphorylation; auxin-enhanced PM H^+^-ATPase activity lowers the pH of the cell wall, activates pH-sensitive enzymes and proteins within the cell wall, and initiates cell-wall loosening and extension growth. Auxin can also increase the quantity of certain PM ATPases by influencing protein expression. This results not only in enhanced H^+^ extrusion, but also eventually in the restoration of the number of PM H^+^-ATPases in a given membrane area.

Root growth, including primary root elongation and root hair formation, is important for the uptake and use of phosphorus (Shen *et al.*, 2006). Optimal primary root elongation or root hair formation requires fine regulation of root tip H^+^ secretion by PM H^+^-ATPase (Palmgren, 2001; Staal *et al.*, 2011; Haruta & Sussmann, 2012). Acidification of the aqueous fraction of the cell wall apoplast by H^+^ excretion via PM H^+^-ATPase is a critical growth-promoting event and a key factor in triggering elongation of the primary root (Moloney, 1981). Root hair development in Arabidopsis is associated with alkalinization of the cytoplasm and acidification of the cell wall, and these processes are regulated by PM H^+^-ATPase-independent H^+^ influx and efflux (Bibikova *et al.*, 1998). However, little is known regarding the role of PM H^+^-ATPases in mediating root H^+^ flux under LP. Thus, in this study, we investigated the involvement and function of different Arabidopsis PM H^+^-ATPase genes in root H^+^ flux in response to LP.

## Materials and methods

### Plant materials, growth conditions, and stress treatment

In this study, the wild-type (WT) Arabidopsis strain used was ecotype Col-0, unless otherwise indicated. The TFT7-overexpressing Arabidopsis plants (denoted OE-TFT7) used in this study was described in a previous publication (Xu *et al.*, 2012). The PM H^+^-ATPase-deficient mutant Arabidopsis plants (*aha1-1*: Salk_065288; *aha1-2*: Salk_118350; *aha2-1*: Salk_062371; *aha2-2*: Salk_082786; *aha7-1*: Salk_042485; *aha7-2*: Salk_056487) were obtained from the Arabidopsis Biological Resource Center (ABRC, Ohio State University, Columbus, OH, USA); the homozygous Arabidopsis mutant was identified by PCR using primers specific to the insertion T-DNA (see Supplementary Table S1 at *JXB* online). To generate double-transgenic Arabidopsis plants, knock-out mutants (*aha2*-2 and *aha7-1*) were crossed with OE-TFT7 plants. Homozygous double-transgenic plants were selected on the basis of PCR with specific primers and kanamycin resistance.

Before planting, Arabidopsis seeds were surface-sterilized with 70% ethanol for 1 min, then with 1% sodium hypochlorite solution plus sodium dodecyl sulfate for 9 min, and subsequently rinsed with sterile deionized water six times. Seeds were then kept in the dark for 3 days at 4 °C for stratification and grown hydroponically using a sugar-free agar medium-based culture system, as described by Xu and Shi (2008). Sterilized seeds were sown on a sucrose-free medium containing full-strength Murashige and Skoog nutrients and 6 g L^–1^ agar. This sugar-free agar medium was dispensed into bottom-removed Eppendorf tubes, which were held in a plastic platform. Two to three seeds were sown in each tube. Seedlings were thinned to one plant per tube after 7 days of growth. The nutrient solution consisted of: 5 mM KNO_3_, 1 mM KH_2_PO_4_, 2 mM MgSO_4_, 2 mM Ca(NO_3_)_2_, 0.5 mM Fe-Na-EDTA, 70 μM H_3_BO_3_, 14 μM MnCl_2_, 0.5 μM CuSO_4_, 1 μM ZnSO_4_, 0.2 μM Na_2_MoO_4_, 10 μM NaCl, and 0.01 μM CoCl_2_. The pH of the solution was adjusted to 6.0 every day and the solution renewed every 2 days. Low-phosphorous (1 μM) conditions were achieved in the nutrient solution by replacing 1 mM KH_2_PO_4_ with 0.999 mM KCl and 0.001 mM KH_2_PO_4_. Arabidopsis plants were grown at 22 ± 1 °C, with a light intensity of 120 μmol photons m^–2^ s^–1^, a 16 h light/8 h dark photoperiod, and a relative humidity of 70% in the growth chamber (Sanyo Electric Co., Ltd, Kyoto, Japan). Twenty-two-day-old Arabidopsis plants were treated with 1 mM phosphorus (control) or 1 μM phosphorus (LP: low-phosphorus stress) for 8 days within the hydroponic system. Control plants and LP-treated plants were harvested at 0-, 1-, 2-, 4-, 6-, and 8-day time points. After harvesting, Arabidopsis plants were frozen immediately in liquid nitrogen and stored at –80 °C until analysis.

### Real-time RT-PCR

Real-time RT-PCR was performed according to the method described by Xu and Shi (2006). Total RNA was extracted from Arabidopsis plants under control and LP conditions. Gene-specific primers for real-time RT-PCR were designed using Primer 5 software (see Supplementary Table S1). *At-ACT2* is a strongly and constitutively expressed ‘housekeeping’ gene in Arabidopsis plants; the mRNA expression level of this gene was designated to be 100 relative expression units (REU). Expression levels of the genes of interest are described relative to *At-ACT2*, specifically, as the ratio of the copy number of the cDNA of interest to the copy number of *At-ACT2* multiplied by 100 REU.

### Measurement of root growth and phosphorus concentration

Root growth was measured according to the method of Xu *et al.* (2013*b*). Root surface area was measured using a root analysis instrument (WinRHIZO; Regent Instruments Inc., Quebec, ON, Canada). The primary root elongation rate of Arabidopsis (μm h^–1^) was calculated based on the displacement of the primary root apex relative to the primary root length over the duration of the experiment. Root hairs in the region 0–5000 μm from the root cap junction were identified in Arabidopsis plants using confocal laser scanning microscopy (Olympus FV-1000 spectral type SPD mar/G/R IX81 Fluoview laser confocal system). Average root hair densities in 100 × 100 μm^2^ sections of the 0–5000 μm region were calculated. The phosphorus concentrations of Arabidopsis roots were determined colorimetrically by the phosphovanadate method (Hanson, 1950) after digestion in a mixture of HNO_3_, HClO_4_, and H_2_SO_4_ (3:1:1, v/v).

### Analysis of proton extrusion

The proton extrusion rate of Arabidopsis roots was analysed following the method of Jin *et al.* (2009). Briefly, 22-day-old Arabidopsis plants were treated with 1 mM phosphorus (control) or 1 μM phosphorus (LP) for 8 days. Arabidopsis plants were transferred to 100-mL pots filled with 1 mM phosphorus solution or 1 μM phosphorus solution. After 12 h, each solution was filtered, and the pH was adjusted to 6.0. The quantity of protons excreted by roots was determined by titrating the solution with 1 mM NaOH.

### PM H^+^-ATPase activity and H^+^ flux in the root tip of Arabidopsis plants

PM H^+^-ATPase activity was determined according to the method described by Xu *et al.* (2012). H^+^ fluxes were measured non-invasively using the scanning ion-selective electrode technique (SIET; SIET system BIO-003A; Younger USA Science and Technology Corp.; Applicable Electronics Inc.; Science Wares Inc., Falmouth, MA, USA), also according to the method described by Xu *et al.* (2012). Under normal growth conditions (control) or low-phosphorus stress (LP), proton (H^+^) flux was measured along the root tip of Arabidopsis plants in the following zones: meristem zone (0–200 μm from the root cap junction), transition zone (200–520 μm from the root cap junction), elongation zone (520–850 μm from the root cap junction), and root hair zone (850–1500 μm from the root cap junction).

## Results

### Expression of Arabidopsis PM H^+^-ATPase genes in response to LP

**Fig. 1.**
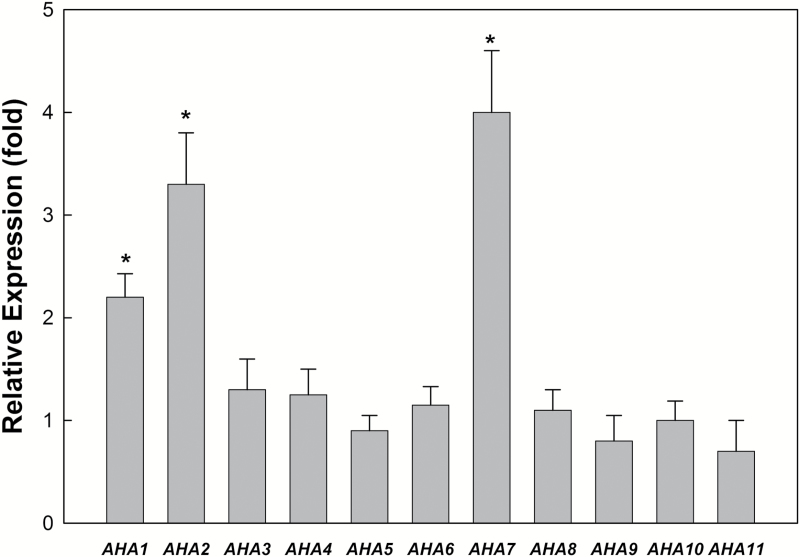
Expression of PM H^+^-ATPase gene family members (*AHA1–AHA11*) in Arabidopsis plants in response to low phosphorus stress. Relative expression of various *AHA* genes in the whole plants of Arabidopsis grown in phosphorus-sufficient conditions (Control; 1 mM) or low phosphorus stress (LP; 1 μM) for 4 days. Values are first normalized to *At-ACT2* (housekeeping gene; 100 REU) and then to control (+P) plants. Expression of these studied genes (*AHA1–AHA11*) under control was taken as 1-fold, and the expression level of these studied genes under LP is shown in this figure. The values are the means and SD of six replicates from two independent experiments. Changes in the relative expression levels of gene were checked for statistical significance according to Student’s *t*-test (*P<*0.05).

**Fig. 2.**
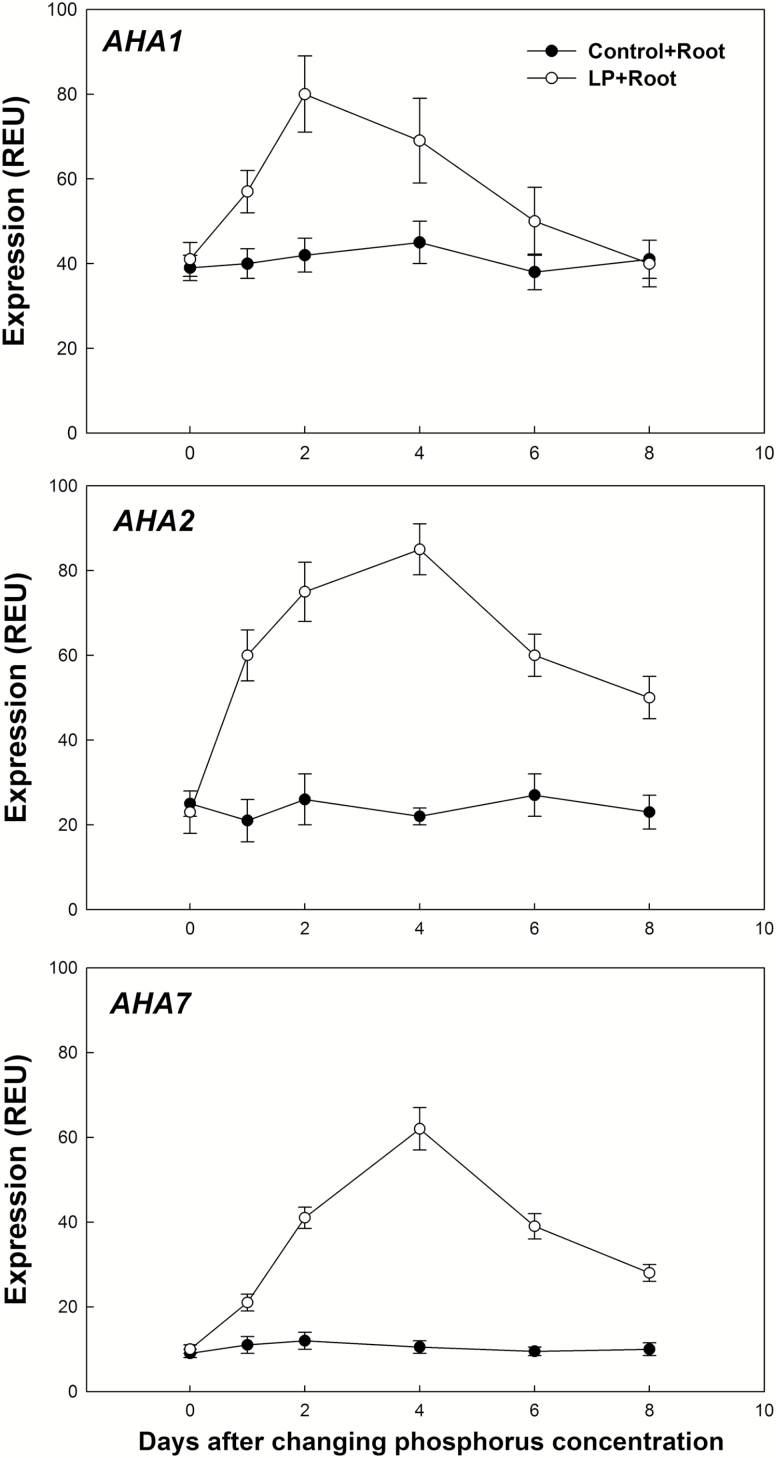
Expression of PM H^+^-ATPase gene family members (*AHA1*, *AHA2*, and *AHA7*) in the roots of Arabidopsis plants under phosphorus sufficient conditions (Control; 1 mM) or low phosphorus stress (LP; 1 μM) over 0, 1, 2, 4, 6, and 8 days. Relative expression levels were calculated and normalized with respect to *At-ACT2* mRNA (=100 REU). Changes in the relative expression levels (REU) of gene mRNA were checked for statistical significance according to Student’s *t*-test (*P<*0.05). The values are the means and SD of six replicates from two independent experiments.

To investigate whether Arabidopsis PM H^+^-ATPase gene family members are regulated by soil phosphorus levels, we examined the expression of PM H^+^-ATPase genes (*AHA1*–*AHA11*) under LP conditions using real-time RT-PCR. To avoid biased results, real-time RT-PCR data are typically expressed relative to the expression of a housekeeping gene (i.e. *At-ACT2*) as an internal control. Using real-time RT-PCR, expression patterns of the 11 PM H^+^-ATPase genes were analysed in whole Arabidopsis plants (leaf and root) under phosphorous-sufficient conditions (control) or low-phosphorus stress (LP) for 4 days (Fig. 1). Under LP, the steady state transcript levels of most PM H^+^-ATPase gene family members appeared relatively unchanged relative to the control condition, but significant differences in three genes, *AHA1*, *AHA2*, and *AHA7*, were observed. The expression of selected PM H^+^-ATPase genes (*AHA1*, *AHA2*, and *AHA7*) was further investigated in Arabidopsis roots under control or LP conditions over 0, 1, 2, 4, 6, and 8 days (Fig. 2). During LP, from day 0 to day 4, the expression of *AHA1*, *AHA2*, and *AHA7* in the roots increased approximately 2-, 4-, and 5-fold, respectively, compared with the control condition. After LP for 8 days, the expression level of *AHA2* and *AHA7* in the roots was approximately 2.5-fold that of the phosphorus-sufficient plants, but no significant change was found in the expression of *AHA1* in roots over this time period.

### Response of PM H^+^-ATPase-deficient mutant roots to LP

**Fig. 3.**
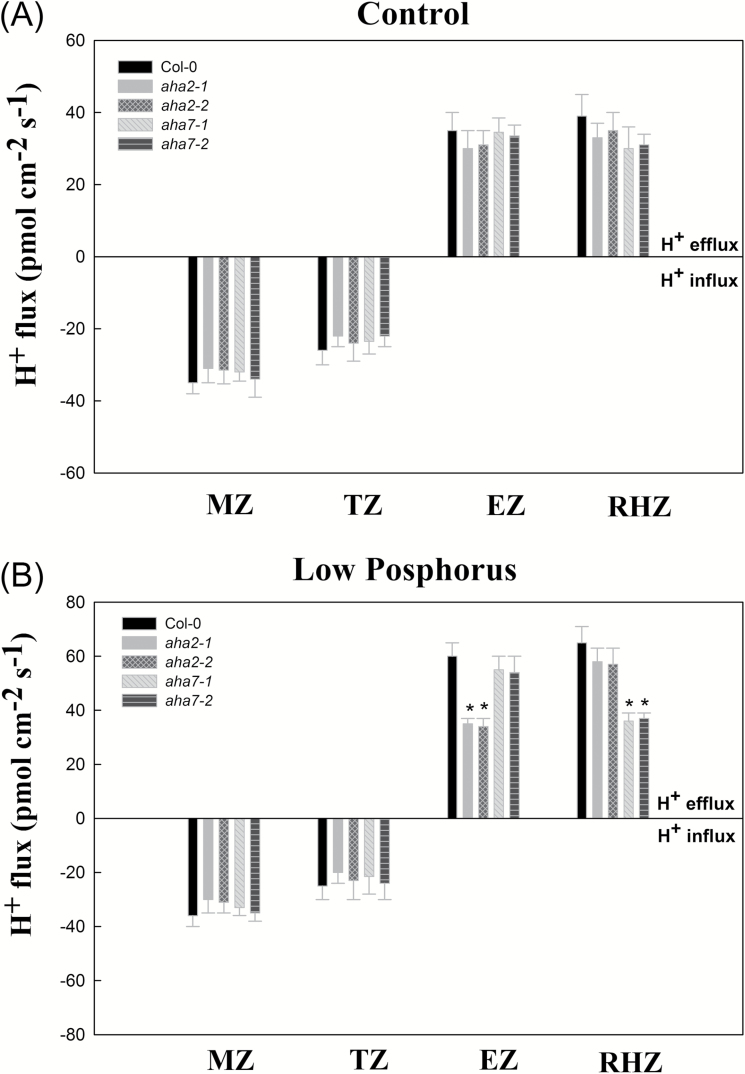
Proton (H^+^) flux in meristem zone (MZ), transition zone (TZ), elongation zone (EZ), and root hair zone (RHZ) in the root tip of wild-type Arabidopsis (Col-0), *aha2* mutant lines (*aha2-1* and *aha2-2*) and *aha7* mutant lines (*aha7-1* and *aha7-2*) under normal growth condition (Control; A) or low phosphorus stress (B). Arabidopsis plants were treated as described in Table 1.

PM H^+^-ATPases play an important role not only in nutrient uptake by roots, but also in root adaption to environmental stress (Desnos, 2008; Xu *et al.*, 2013*a*). We therefore analysed root phosphorus content, root surface area, primary root elongation, root hair density, PM H^+^-ATPase activity, proton extrusion, and proton flux in the roots of Col-0 (WT Arabidopsis plant), *aha1* mutant lines (*aha1-1* and *aha1-2*), *aha2* mutant lines (*aha2-1* and *aha2-2*), and *aha7* mutant lines (*aha7-1* and *aha7-2*) under normal growth conditions (control) and LP (Table 1 and Fig. 3). As shown in Table 1A, there was no significant difference in phosphorus content between the *AHA*-deficient mutants and Col-0 under control conditions. Under LP, no significant change was found in the phosphorus content of *aha1* mutant lines compared with Col-0; however, the phosphorus contents of *aha2* and *aha7* mutant lines were significantly lower than that of Col-0. As shown in Table 1B, there was no significant difference in the root surface area between AHA-deficient mutants and Col-0 under control conditions. Under LP, no significant change was found in the root surface area of *aha1* mutant lines compared with Col-0; however, the root surface areas of *aha2* and *aha7* mutant lines were significantly lower than that of Col-0. As shown in Table 1C, there was no significant difference in primary root elongation between AHA-deficient mutants and Col-0 under control conditions. Under LP, no significant change was found in the primary root elongation of *aha1* or *aha7* mutant lines compared with Col-0; however, the primary root elongation of *aha2* mutant lines was significantly lower than that of Col-0. As shown in Table 1D, there was no significant difference in root hair density between AHA-deficient mutants and Col-0 under control conditions. Under LP, no significant change was found in root hair density of *aha1* or *aha2* mutant lines compared with Col-0; however, the root hair density of *aha7* mutant lines was significantly lower than that of Col-0. As shown in Table 1E, there was no significant difference in PM H^+^-ATPase activity between AHA-deficient mutants and Col-0 under control conditions. Under LP, no significant change was found in PM H^+^-ATPase activity of *aha1* mutant lines compared with Col-0; however, the PM H^+^-ATPase activities of *aha2* and *aha7* mutant lines were significantly lower than that of Col-0. As shown in Table 1F, there was no significant difference in proton extrusion between AHA-deficient mutants and Col-0 under control condition. Under LP, no significant change was found in proton extrusion in *aha1* mutant lines compared with Col-0; however, proton extrusion was significantly lower in *aha2* and *aha7* mutant lines relative to Col-0. We also investigated proton (H^+^) flux in the meristem zone (MZ), transition zone (TZ), elongation zone (EZ), and root hair zone (RHZ) in the root tip of WT Arabidopsis (Col-0), *aha2* mutant lines (*aha2-1* and *aha2-2*), and *aha7* mutant lines (*aha7-1* and *aha7-2*) under normal growth conditions (control) and LP. As shown in Fig. 3, there was no significant difference in proton flux between AHA-deficient mutants and Col-0 under control conditions. Under LP, there was no significant difference in proton flux in the MZ and TZ of AHA-deficient mutants and Col-0. However, under LP, proton efflux in the EZ of *aha2* mutant lines was significantly lower than in the EZ of Col-0 and *aha7*. Finally, under LP, proton efflux in the RHZ of *aha7* mutant lines was significantly lower than in the EZ of Col-0 and *aha2*.

**Table 1.** Phosphorus content, root surface area, elongation rate of primary root, root hair density, activity of PM H^+^-ATPase and proton extrusion in the roots of Col-0 (wild-type Arabidopsis plant), aha1 mutant lines (aha1-1 and aha1-2), aha2 mutant lines (aha2-1 and aha2-2) and aha7 mutant lines (aha7-1 and aha7-2) under normal growth condition (Control) or low phosphorus stress (LP) Twenty-two-day-old Arabidopsis plants (wild-type and mutant lines) were treated with 1 mM phosphorus (Control) and 1 μM phosphorus (LP) for 8 days under hydroponic system. Then, 30-day-old Arabidopsis plants were used for experimental analysis. The data were subjected to analysis of variance and *post hoc* comparisons were performed with Duncan’s multiple range test at the *P<*0.05 level. The statistical software program used was SPSS version 13.0. The values are the means and SD of six replicates from two independent experiments. Values with the same letter (a or b or c) are not significantly different at *P<*0.05 level under the same treatment (control or LP).

	Col-0	*aha1-1*	*aha1-2*	*aha2-1*	*aha2-2*	*aha7-1*	*aha7-2*
(A) Root phosphorus content (μg plant^–1^)							
Control	9.2 ± 0.3 a	8.9 ± 0.5 a	9.1 ± 0.2 a	9.0 ± 0.5 a	8.8 ± 0.6 a	9.3 ± 0.2 a	9.0 ± 0.3 a
LP	6.9 ± 0.5 a	6.3 ± 0.2 a	6.5 ± 0.5 a	5.1 ± 0.3 b	5.0 ± 0.4 b	5.2 ± 0.2 b	4.9 ± 0.6 b
(B) Root surface area (cm^2^ plant^–1^)							
Control	6.6 ± 0.5 a	6.0 ± 0.4 a	6.1 ± 0.3 a	6.3 ± 0.4 a	5.9 ± 0.3 a	6.5 ± 0.6 a	6.2 ± 0.2 a
LP	4.6 ± 0.3 a	4.4 ± 0.2 a	4.5 ± 0.3 a	3.1 ± 0.2 b	3.0 ± 0.3 b	3.3 ± 0.1 b	3.2 ± 0.2 b
(C) Elongation rate of primary root (μm h^–1^)							
Control	120 ± 8.1 a	116 ± 9.0 a	115 ± 7.5 a	110 ± 9.3 a	109 ± 8.2 a	118 ± 8.5 a	115 ± 9.4 a
LP	90 ± 6.2 a	88 ± 5.5 a	91 ± 9.6 a	61 ± 8.1 b	66 ± 7.3 b	85 ± 8.5 a	80 ± 9.8 a
(D) Root hair density (no. in 100 × 100 mm^2^)							
Control	1.35 ± 0.11 a	1.30 ± 0.12 a	1.34 ± 0.09 a	1.31 ± 0.15 a	1.32 ± 0.09 a	1.22 ± 0.09 a	1.18 ± 0.14 a
LP	1.98 ± 0.15 a	1.91 ± 0.12 a	1.89 ± 0.11 a	1.75 ± 0.20 a	1.78 ± 0.18 a	1.32 ± 0.14 b	1.30 ± 0.17 b
(E) Activity of PM H+-ATPase (μmol min^–1^ mg^–1^ protein)							
Control	1.28 ± 0.09 a	1.21 ± 0.13 a	1.22 ± 0.18 a	1.10 ± 0.15 a	1.12 ± 0.19 a	1.18 ± 0.14 a	1.19 ± 0.12 a
LP	2.32 ± 0.13 a	2.06 ± 0.15 a	2.02 ± 0.11 a	1.25 ± 0.12 b	1.18 ± 0.18 b	1.46 ± 0.13 b	1.48 ± 0.15 b
(F) Rate of root proton extrusion (μmol h^–1^ 10 g^–1^ FW)							
Control	1.6 ± 0.3 a	1.4 ± 0.2 a	1.3 ± 0.2 a	1.1 ± 0.3 a	1.2 ± 0.2 a	1.4 ± 0.1 a	1.4 ± 0.3 a
LP	2.9 ± 0.3 a	2.7 ± 0.1 a	2.6 ± 0.2 a	1.3 ± 0.2 b	1.2 ± 0.1 b	1.7 ± 0.2 b	1.7 ± 0.1 b

### Effect of 14-3-3 proteins on the root response of PM H^+^-ATPase-deficient mutants to LP

**Fig. 4.**
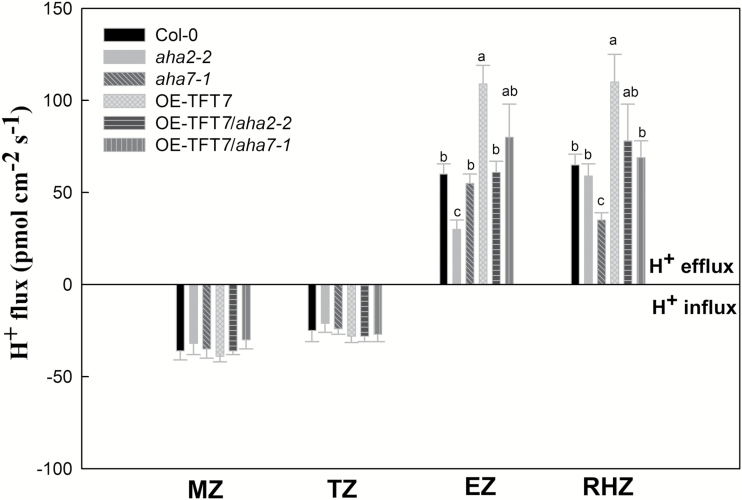
Proton (H^+^) flux in meristem zone (MZ), transition zone (TZ), elongation zone (EZ) and root hair zone (RHZ) in the root tip of wild-type Arabidopsis (Col-0), OE-TFT7 (TFT7-overexpressing Arabidopsis plants) and mutant lines (*aha2-2*, *aha7-1*, OE-TFT7/*aha2-2* and OE-TFT7/*aha7-1*) in response to low phosphorus stress. Arabidopsis plants were treated as described in Table 1.

In our previous study (Xu *et al.*, 2012), we demonstrated that the tomato 14-3-3 protein TFT7 directly functions in the root by enhancing Arabidopsis H^+^ secretion under LP. Subsequently, we set out to establish whether AHA2 or AHA7 is involved in the root response of TFT7-overexpressing Arabidopsis plants (OE-TFT7) to LP. By interbreeding transgenic plant strains, we obtained OE-TFT7 plants that lacked *AHA2* or *AHA7*. Root phosphorus content, root surface area, primary root elongation, root hair density, PM H^+^-ATPase activity, proton extrusion, and proton flux were analysed in the roots of Col-0 (WT Arabidopsis plant), OE-TFT7 (TFT7-overexpressing Arabidopsis plants), and mutant lines (*aha2-2*, *aha7-1*, OE-TFT7/*aha2-2*, and OE-TFT7/*aha7-1*) under normal growth conditions (control) and LP (Table 2 and Fig. 4). As shown in Table 2, there was no significant difference in the phosphorus content among these plants (Col-0, OE-TFT7, *aha2-2*, *aha7-1*, OE-TFT7/*aha2-2*, and OE-TFT7/*aha7-1*) under control conditions. Under LP, the phosphorus contents, root surface areas, primary root elongation, of Col-0, OE-TFT7/*aha2-2*, and OE-TFT7/*aha7-1* (no significance difference was found among these strains) were significantly lower than that of OE-TFT7. Furthermore, the primary root elongation of OE-TFT7/*aha7-1* was significantly higher than that of *aha2-2*, but no significant difference was observed between OE-TFT7/*aha7-1* and Col-0, *aha7-1*, OE-TFT7/*aha2-2*, and OE-TFT7. Under LP, the root hair densities of Col-0, *aha2-2*, and OE-TFT7/*aha7-1* (no significance difference was found among these strains) were significantly higher than that of *aha7-1*, but lower than that of OE-TFT7. In addition, the root hair density of OE-TFT7/*aha2-2* was significantly higher than that of *aha7-1*; however, no significant difference was found in the root hair density of OE-TFT7/*aha2-2* compared with Col-0, *aha2-2*, OE-TFT7/*aha7-1*, and OE-TFT7. As shown in Table 2E, F, there was no significant difference in PM H^+^-ATPase activity or proton extrusion among Col-0, *aha2-2*, *aha7-1*, OE-TFT7/*aha2-2*, and OE-TFT7/*aha7-1* under control conditions; however, H^+^-ATPase activity and proton extrusion in each of these strains was significantly lower than in OE-TFT7. Under LP, PM H^+^-ATPase activity and proton extrusion in Col-0, OE-TFT7/*aha2-2*, and OE-TFT7/*aha7-1* (no significance difference was found among these strains) was significantly higher than in *aha2-2* or *aha7-1*, but lower than in OE-TFT7. As shown in Fig. 4, under LP, there was no significant difference in proton flux among these plants (Col-0, OE-TFT7, *aha2-2*, *aha7-1*, OE-TFT7/*aha2-2*, and OE-TFT7/*aha7-1*) in the MZ or TZ of the root tip. However, in the EZ of the root tip, proton efflux in Col-0, *aha7-1*, and OE-TFT7/*aha2-2* (no significance difference was found among these strains) was significantly higher than in *aha2-2*, but lower than in OE-TFT7. Proton efflux in OE-TFT7/*aha7-1*, however, was significantly higher than in *aha2-2*, but no significant change was found in OE-TFT7/*aha7-1* compared with Col-0, *aha7-1*, OE-TFT7/*aha2-2*, and OE-TFT7. Further, in the RHZ of the root tip, proton efflux in Col-0, *aha2-2*, and OE-TFT7/*aha7-1* (no significance difference was found among these strains) was significantly higher than in *aha7-1*, but lower than in OE-TFT7. Proton efflux in OE-TFT7/*aha2-2* was significantly higher than in *aha7-1*, but no significant change was found in OE-TFT7/*aha2-2* compared with Col-0, *aha2-2*, OE-TFT7/*aha7-1*, and OE-TFT7.

**Table 2.** *Phosphorus content, root surface area, elongation rate of primary root, root hair density, activity of PM H* ^*+*^ *-ATPase and proton extrusion in the roots of Col-0 (wild-type Arabidopsis plant), OE-TFT7 (TFT7-overexpressing Arabidopsis plants) and mutant lines* (aha2-2, aha7-1*, OE-TFT7/*aha2-2 *and OE-TFT7/*aha7-1*) under normal growth condition (Control) or low phosphorus stress (LP*) Twenty-two-day-old Arabidopsis plants (wild-type, TFT7-overexpressing and mutant lines) were treated with 1 mM phosphorus (Control) and 1 μM phosphorus (LP) for 8 days under hydroponic system. Then, 30-day-old Arabidopsis plants were used for experimental analysis. The data were subjected to analysis of variance and *post hoc* comparisons were done with Duncan’s multiple range test at the *P<*0.05 level. The statistical software program used was SPSS version 13.0. The values are the means and SD of six replicates from two independent experiments. Values with the same letter (a, b or c) are not significantly different at *P<*0.05 level under the same treatment (control or LP).

	Col-0	*aha2-2*	*aha7-1*	OE-TFT7	OE-TFT7/*aha2-2*	OE-TFT7/*aha7-1*
(A) Root phosphorus content (μg plant^–1^)						
Control	9.1 ± 0.2 a	9.0 ± 0.4 a	8.9 ± 0.6 a	9.5 ± 0.5 a	9.1 ± 0.2 a	9.0 ± 0.4 a
LP	6.8 ± 0.2 a	5.1 ± 0.2 b	5.0 ± 0.4 b	8.3 ± 0.2 c	7.2 ± 0.3 a	7.0 ± 0.5 a
(B) Root surface area (cm^2^ plant^–1^)						
Control	6.5 ± 0.4 a	6.6 ± 0.5 a	6.4 ± 0.3 a	6.8 ± 0.6 a	6.3 ± 0.2 a	6.2 ± 0.1 a
LP	4.4 ± 0.2 a	3.1 ± 0.2 b	3.2 ± 0.3 b	5.9 ± 0.3 c	4.6 ± 0.2 a	4.5 ± 0.3 a
(C) Elongation rate of primary root (μm h^–1^)						
Control	122 ± 7.1 a	117 ± 8.5 a	115 ± 9.4 a	129 ± 9.2 a	116 ± 9.0 a	113 ± 9.8 a
LP	91 ± 5.9 a	63 ± 6.1 b	83 ± 9.5 a	118 ± 5.2 c	93 ± 8.0 a	101 ± 13.7 ac
(D) Root hair density (no. in 100 × 100 mm^2^)						
Control	1.36 ± 0.15 a	1.32 ± 0.10 a	1.26 ± 0.16 a	1.49 ± 0.25 a	1.36 ± 0.09 a	1.30 ± 0.14 a
LP	1.88 ± 0.12 a	1.80 ± 0.16 a	1.30 ± 0.12 b	2.38 ± 0.12 c	2.12 ± 0.21 ac	1.98 ± 0.14 a
(E) Activity of PM H^+^-ATPase (μmol min^–1^ mg^–1^ protein)						
Control	1.25 ± 0.11 a	1.15 ± 0.15 a	1.20 ± 0.16 a	1.6 ± 0.10 b	1.28 ± 0.13 a	1.29 ± 0.17 a
LP	2.30 ± 0.15 a	1.19 ± 0.11 b	1.45 ± 0.17 b	3.17 ± 0.14 c	2.46 ± 0.22 a	2.51 ± 0.25 a
(F) Rate of root proton extrusion (μmol h^–1^ 10 g^–1^ FW)						
Control	1.5 ± 0.4 a	1.3 ± 0.2 a	1.4 ± 0.1 a	2.5 ± 0.3 b	1.7 ± 0.2 a	1.6 ± 0.3 a
LP	2.8 ± 0.3 a	1.5 ± 0.1 b	1.6 ± 0.2 b	5.0 ± 0.6 c	3.0 ± 0.3 a	3.1 ± 0.4 a

### Proton regulation and auxin modulation in the root response of PM H^+^-ATPase-deficient mutants to LP

**Fig. 5.**
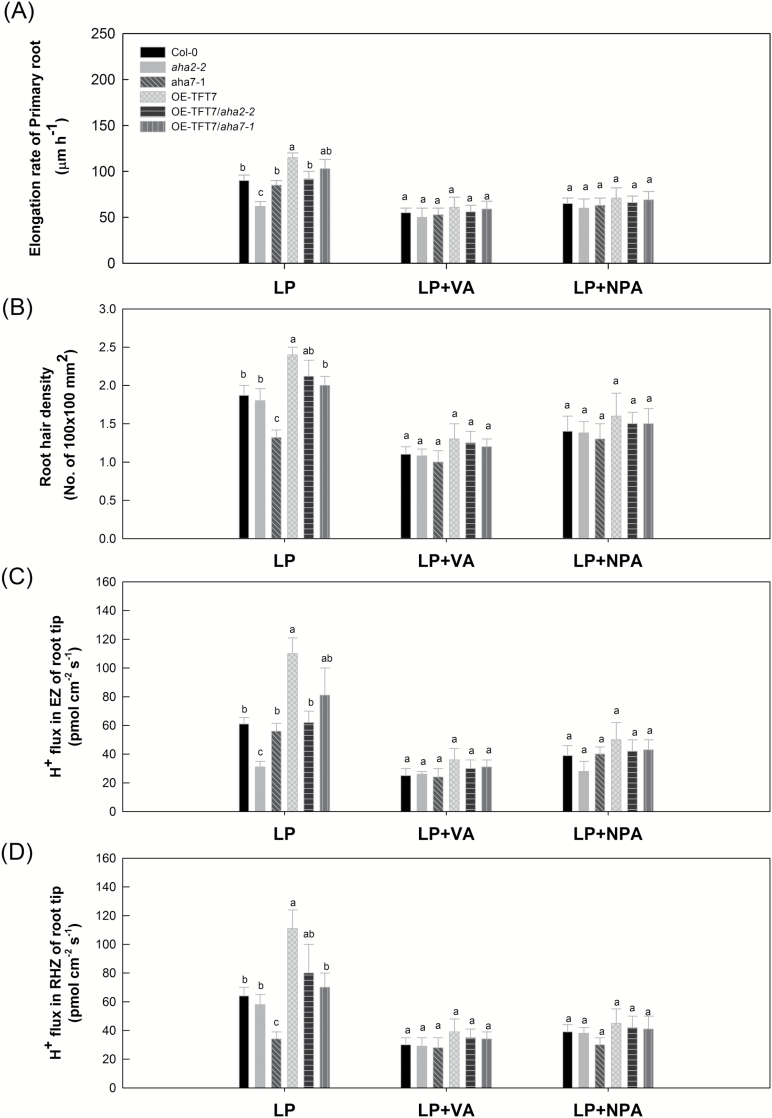
Root response of wild-type Arabidopsis plants (Col-0), OE-TFT7 (TFT7-overexpressing Arabidopsis plants) and mutant lines (*aha2-2*, *aha7-1*, OE-TFT7/*aha2-2* and OE-TFT7/*aha7-1*) to low phosphorus stress. Twenty-two-day-old Arabidopsis plants were treated with 1 μM phosphorus (LP), LP with 1 mM vanadate (VA; PM H^+^-ATPase inhibitor) or NPA (auxin flux inhibitor; 10 μM) for 8 days under a hydroponic system. (A) Elongation rate of primary root; (B) root hair density in the root tip of Arabidopsis (0–5000 μm from the root cap junction); (C) H^+^ flux in elongation zone (EZ); and (D) H^+^ flux in root hair zone (RHZ) in the root tip of Arabidopsis plants.

Our previously reported data (Xu *et al.*, 2013*b*) demonstrate that auxin plays important roles in proton flux in the root tip in response to environmental stress. In the study described here, we investigated the root response of WT Arabidopsis plants (Col-0), OE-TFT7 (TFT7-overexpressing Arabidopsis plants), and mutant lines (*aha2-2*, *aha7-1*, OE-TFT7/*aha2-2*, and OE-TFT7/*aha7-1*) under LP, and LP with vanadate (VA; a PM H^+^-ATPase inhibitor) or *N*-1-naphthylphthalamic acid (NPA; an auxin flux inhibitor). Under LP, primary root elongation in Col-0, *aha7-1*, OE-TFT7, OE-TFT7/*aha2-2*, and OE-TFT7/*aha7-1* was significantly higher than in *aha2-2*. However, under LP with VA or NPA, no significant difference in primary root elongation was found among these strains (Fig. 5A). Although the root hair densities of Col-0, *aha2-2*, OE-TFT7, OE-TFT7/*aha2-2*, and OE-TFT7/*aha7-1* were significantly higher than that of *aha7-1* under LP, no significant difference was found among them under LP with VA or NPA (Fig. 5B). H^+^ flux in the root tip EZ in Col-0, *aha7-1*, OE-TFT7, OE-TFT7/*aha2-2*, and OE-TFT7/*aha7-1* was significantly higher than in *aha2-2* under LP; however, under LP with VA or NPA, no significant differences were found in H^+^ flux in the root tip EZ among strains (Fig. 5C). Under LP, although H^+^ flux in the root tip RHZ in Col-0, *aha2-2*, OE-TFT7, OE-TFT7/*aha2-2*, and OE-TFT7/*aha7-1* was significantly higher than in *aha7-1*, no significant difference was found among them under LP with VA or NPA (Fig. 5B).

## Discussion

### AHA2 plays a regulatory role in H^+^ efflux in the root elongation zone in the context of primary root elongation in response to LP

One of the most pronounced adaptations of plants to low-phosphorus soil conditions is the enhancement of root elongation (Richardson, 2009). Primary root elongation, regulated by a sensory zone in the root tip, plays an important role in the plastic acclimation response to fluctuating soil environments (Baluska *et al.*, 2010). PM H^+^-ATPase-regulated proton (H^+^) excretion has an important function in this process. The acid growth theory implicates that apoplastic H^+^ is the major wall-loosening factor that causes root cell extension (Staal *et al.*, 2011). Apoplastic H^+^ is mainly attributed to H^+^ efflux mediated by PM H^+^-ATPase (Palmgren, 2001). Wang *et al.* (2014) proposed that *AHA2* is useful for promoting plant growth by modulating light-induced stomatal opening. AHA2 is also important in maintaining the plasma membrane proton motive force (PMF) during plant seedling growth (Haruta and Sussman, 2012). We found that the expression of *AHA2* is up-regulated in roots under LP (Figs 1 and 2). As shown in Table 1, when *AHA2* is deficient in Arabidopsis plants, as in the *aha2* mutant, root phosphorus content, root surface area, PM H^+^-ATPase activity, and root proton extrusion are lower than in the Col-0 (WT) and the *aha1* mutant. Although there was no significant difference in root hair density between *aha2* and Col-0, primary root elongation was lower in *aha2* than in Col-0 and *aha7*. Further, under LP, although no significant difference in H^+^ efflux of the root hair zone was found between Col-0 and *aha2*, H^+^ efflux in the root elongation zone of *aha2* was significantly lower than that in Col-0 (Fig. 3). Thus, our results suggest that *AHA2* plays a regulatory role in H^+^ efflux in the root elongation zone in the context of the primary root elongation response to LP.

### AHA7-H^+^-mediated efflux in the root hair zone is important for root hair formation under LP

Plants have developed physiological and morphological adaptations to acquire phosphorus from the soil. These adaptations include not only increased root length for phosphorus uptake over a greater soil volume, but also root hair formation for more efficient acquisition of phosphorus (Raghothama, 1999; Hammond *et al.*, 2004; Lambers *et al.*, 2006). Studies have shown that PM H^+^-ATPase-regulated H^+^ flux is important for root hair development (Palmgren, 2001). For instance, PM H^+^-ATPase protein is highly concentrated in root hairs, and several isoforms of plant PM H^+^-ATPase localize to root hairs. H^+^ circuits have also been found in root hairs: H^+^ enters the root hair at the root tip and leaves the root hair below this tip. *AHA7* is the PM H^+^-ATPase responsible for iron deficiency-induced differentiation of rhizodermic cells. The action of *AHA7* is not affected by the modulation of Arabidopsis 14-3-3 protein GRF11 under conditions of iron deficiency (Yang *et al.*, 2013). We also found that the expression of *AHA7* is up-regulated in roots under LP (Fig. 1 and Fig. 2). According to Table 1, when *AHA7* is deficient in Arabidopsis plants, as in the *aha7* mutant, root phosphorus content, root surface area, PM H^+^-ATPase activity, and root proton extrusion are lower than in the Col-0 (WT) and *aha1* mutant. Although there was no significant difference in primary root elongation between *aha7* and Col-0, root hair density of *aha7* was lower than that of Col-0 and *aha2*. Further, under LP, although no significant difference was found in H^+^ efflux in the root elongation zone between Col-0 and *aha7*, H^+^ efflux in the root hair zone of *aha7* was significantly lower than that of Col-0 (Fig. 3). Thus, our results suggest that *AHA7*-mediated H^+^ efflux in the root hair zone is important for root hair formation under LP.

### Effect on AHA2 or AHA7 regulation of the action of 14-3-3 proteins or auxin modulation in the root response to LP

In plants, 14-3-3 proteins regulate the activity of PM H^+^-ATPases (Chevalier *et al.*, 2009). The binding of 14-3-3 proteins to PM H^+^-ATPases blocks the autoinhibitory action of the PM H^+^-ATPase C-terminus, thus increasing PM H^+^-ATPase activity. The 14-3-3 binding sites on Arabidopsis were mapped to the extreme C-terminus, where the penultimate threonine is phosphorylated. The plant 14-3-3 protein family has many members, with high levels of conservation, and most can bind PM H^+^-ATPase. Environmental factors, such as light, can stimulate 14-3-3 interactions with PM H^+^-ATPase (Kinoshita and Shimazaki, 1999). This increase in PM H^+^-ATPase activity causes guard cells to swell, opening the stomata. Our previous work showed that the tomato 14-3-3 protein *TFT7* acts mainly in the root and is involved in the local response of plants to LP by activating root plasma membrane H^+^-ATPase (Xu *et al.*, 2012). By interbreeding strains, we obtained TFT7-overexpressing Arabidopsis plants (OE-TFT7) that lacked *AHA2* or *AHA*7. As shown in Table 2, under LP, root phosphorus content, root surface area, PM H^+^-ATPase activity, and root proton extrusion of OE-TFT7/*aha2* and OE-TFT7/*aha7* were significantly lower than that of OE-TFT7. However, primary root elongation in OE-TFT7 was significantly higher than in OE-TFT7/*aha2*, but not OE-TFT7/*aha7*. The root hair densities in OE-TFT7 were significantly higher than in OE-TFT7/*aha7*, but not OE-TFT7/*aha2*. Further, under LP, H^+^ efflux in the root elongation zone of OE-TFT7 was significantly higher than in OE-TFT7/*aha2*, but not OE-TFT7/*aha7*. Conversely, H^+^ efflux in the root hair zone of OE-TFT7 was significantly higher than in OE-TFT7/*aha7*, but not OE-TFT7/*aha2* (Fig. 4). It is well known that plants enhance proton extrusion by up-regulating PM H^+^-ATPases to facilitate phosphorus mobilization and uptake under LP. As shown in Table 2, root proton extrusion of *aha2* or *aha7* was significantly lower than that of Col-0 (wild-type) under LP. And also, when *AHA2* or *AHA7* was deficient in TFT7-overexpressing plants (OE-TFT7), root proton extrusion of OE-TFT7 under LP was decreased greatly. Thus, our results suggest that *AHA2* or *AHA7* is involved in the action of 14-3-3 proteins in the root response to LP.

In plants, auxin also regulates the activity of PM H^+^-ATPases. The acid growth theory implicates (Hager, 2003) that (i) PM H^+^-ATPase is responsible for the extrusion of H^+^ into the cell wall; (ii) auxin enhances the activity of PM H^+^-ATPase, thus causing a lowering of the cell wall pH; (iii) the reduced pH in the cell wall activates cell wall-loosening enzymes and initiates the enlargement of the cell. Our previous results also suggest that auxin modulation and auxin cell–cell transport are key regulators of acidity-induced cell elongation in the root under alkaline stress (Xu *et al.*, 2013*a*). In this study, under LP, primary root elongation and H^+^ efflux in the root elongation zone of OE-TFT7 were significantly higher than in TFT7/*aha2* (Fig. 5). In addition, the root hair density and H^+^ efflux in the root hair zone of OE-TFT7 were significantly higher than in TFT7/*aha7*. Under PM H^+^-ATPase inhibition or auxin flux inhibition, however, no significant difference in these indexes was found among these treated plants (Fig. 5). Thus, our results suggest that the H^+^-mediated regulation of *AHA2* and *AHA7* is affected by auxin modulation in the root response to LP.

In conclusion, H^+^ secretion, regulated by PM H^+^-ATPases, plays an important role in the plant response to LP. On the one hand, H^+^ extrusion in the rhizosphere is important for the release of phosphorus from the soil. On the other hand, H^+^ efflux (cell–cell transport) in the root can loosen the cell wall to promote root cell elongation to further increase phosphorus uptake. In this process, *AHA2* plays a regulatory role in primary root elongation by modulating H^+^ efflux in the root elongation zone in response to LP. Similarly, *AHA7* is important for root hair formation, because it mediates H^+^ efflux in the root hair zone under LP. The H^+^-mediated regulation of *AHA2* or *AHA7* are affected by 14-3-3 proteins and auxin modulation in the root response to LP. We conclude that *AHA2* and *AHA7*, which encode two Arabidopsis plasma membrane H^+^-ATPases, differentially modulate H^+^ efflux in the root tip and thereby have distinct and overlapping roles in response to LP.

## Supplementary Material

Supplementary Data
