# Supplementary material for: Arabidopsis plasma membrane H+-ATPase genes AHA2 and AHA7 have distinct and overlapping roles in the modulation of root tip H+ efflux in response to low-phosphorus stress
Source: J Exp Bot. 2017 Mar 28;68(7):1731–41. doi: 10.1093/jxb/erx040 (PMC5441905; doi:10.1093/jxb/erx040)
Supplement: Supplementary Data [file erx040_Supplementary_Data.zip › supplementary_table_S1.pdf]

Arabidopsis plasma membrane H^+-ATPase genes *AHA2* and *AHA7* have distinct and overlapping roles in the modulation of root tip H^+ efflux in response to low-phosphorus stress

Wei Yuan[a,b,#], Dongping Zhang[a,b,c,#], Tao Song[a,b], Feiyun Xu[a,b,d], Sheng Lin[a,b], Weifeng Xu[a,b,*], Qianfeng Li[c], Yiyong Zhu[d], Jiansheng Liang[c] and Jianhua Zhang[b,*]

Supplementary data

Table S1 Gene specific primers used in PCR for PM ATPase gene family members (*AHA1-AHA11*) or Salk lines in Arabidopsis plants

Gene or line	Primer (5' to 3')	
**Arabidopsis AHA genes:**		
At-ACT2	[F]:ATTCAGATGCCCAGAAGTCTTGTTCC	[R]: ACCACCGATCCAGACACTGTACTTCC
AHA1	[F]: TTGAAGTTTGCCATTCGGTA	[R]: GCCCATTGAGCTTCTCTTTC
AHA2	[F]: TTGTTGAACGTCCTGGAGCA	[R]: AATTCCCAGTTGGCGTAAACC
AHA3	[F]: AGTCGACAGCACAAACCAAG	[R]: CTGTAGTGTCTGCGCTGGAT
AHA4	[F]: ATGTTCACAGATCGAACCCA	[R]: GAAGTTCACGCAACCTAGCA
AHA5	[F]: ATGCGGTTTCAATCACGATA	[R]: TCATGATTGTTCCGTCGTTT
AHA6	[F]: TATCCGATCCAACATCGAAA	[R]: ACAGACAACACAGTGGGCAT
AHA7	[F]: AGAAATAGCGCAACGGAACT	[R]: TTGCAGCTGATTCAACCTTC
AHA8	[F]: CATTTGTGGGATGACTGGAG	[R]: GCAATTCCAATATCCGCTTT
AHA9	[F]: GGCAAGATTTCGTCGGTATT	[R]: CCACTTGCCATCTCTCAGAA
AHA10	[F]: TACGCATTGAGTGGAGAAGC	[R]: GAACGGCTTCCTCTGAGTTC
AHA11	[F]: GGATTTCGGGAAAGAACAAA	[R]: TCTTAGCTTCTTCGGCCATT
Primers for all Salk lines	BP	ATTTTGCCGATTTCGGAAC
Primers specific to the	LP	GCGTTGTAACTCTTGCAGTTTG
T-DNA of Salk_065288	RP	CATCTTCTTTTGGCTGCAGAC
Primers specific to the	LP	TTCGATTCTCCCACACAGATC
T-DNA of Salk_118350	RP	ACGGATTGTGATTGAGACTGC
Primers specific to the	LP	AAAAGAATCGTGTGTGATCGG
T-DNA of Salk_062371	RP	TCAGCCTTCATGGTTCTGATC
Primers specific to the	LP	TTGAAAAGGCTGATGGATTTG
T-DNA of Salk_082786	RP	CTCCAGGACGTTCAACAAAAG
Primers specific to the	LP	GCCTGGTCAATCTTTAAAGGG
T-DNA of Salk_042485	RP	TTTTTCTGGTCCAGGTTTGTG
Primers specific to the	LP	CAGTTGCATGATGTCACATCC
T-DNA of Salk_056487	RP	ACAATCGTTTTAAACCAGGGG
